# Supplementary material for: Magmatism controls global oceanic transform fault topography
Source: Nat Commun. 2024 Mar 1;15:1914. doi: 10.1038/s41467-024-46197-9 (PMC10907720; doi:10.1038/s41467-024-46197-9)
Supplement: Supplementary file 1 — Supplementary Information [file 41467_2024_46197_MOESM1_ESM.pdf]

Supplementary Information for:

Title: Magmatism Controls Global Oceanic Transform Fault Topography

Authors: Xiaochuan Tian[1]*, Mark D. Behn[1], Garrett Ito[2], Jana C. Schierjott[2], Boris J. P. Kaus[3], Anton A. Popov[3]

Affiliations:

[1] Department of Earth and Environmental Sciences, Boston College; Chestnut Hill, MA, US.

[2] Department of Earth Sciences, University of Hawaii; Honolulu, Hawaii, US.

[3] Institute of Geosciences, Johannes Gutenberg University Mainz; Mainz, Germany.

*Corresponding author. Email: x.tian@bc.edu

This PDF file includes:

Supplementary Figures 1 to 6
Supplementary Tables 1 and 2
Supplementary Discussion
Supplementary References

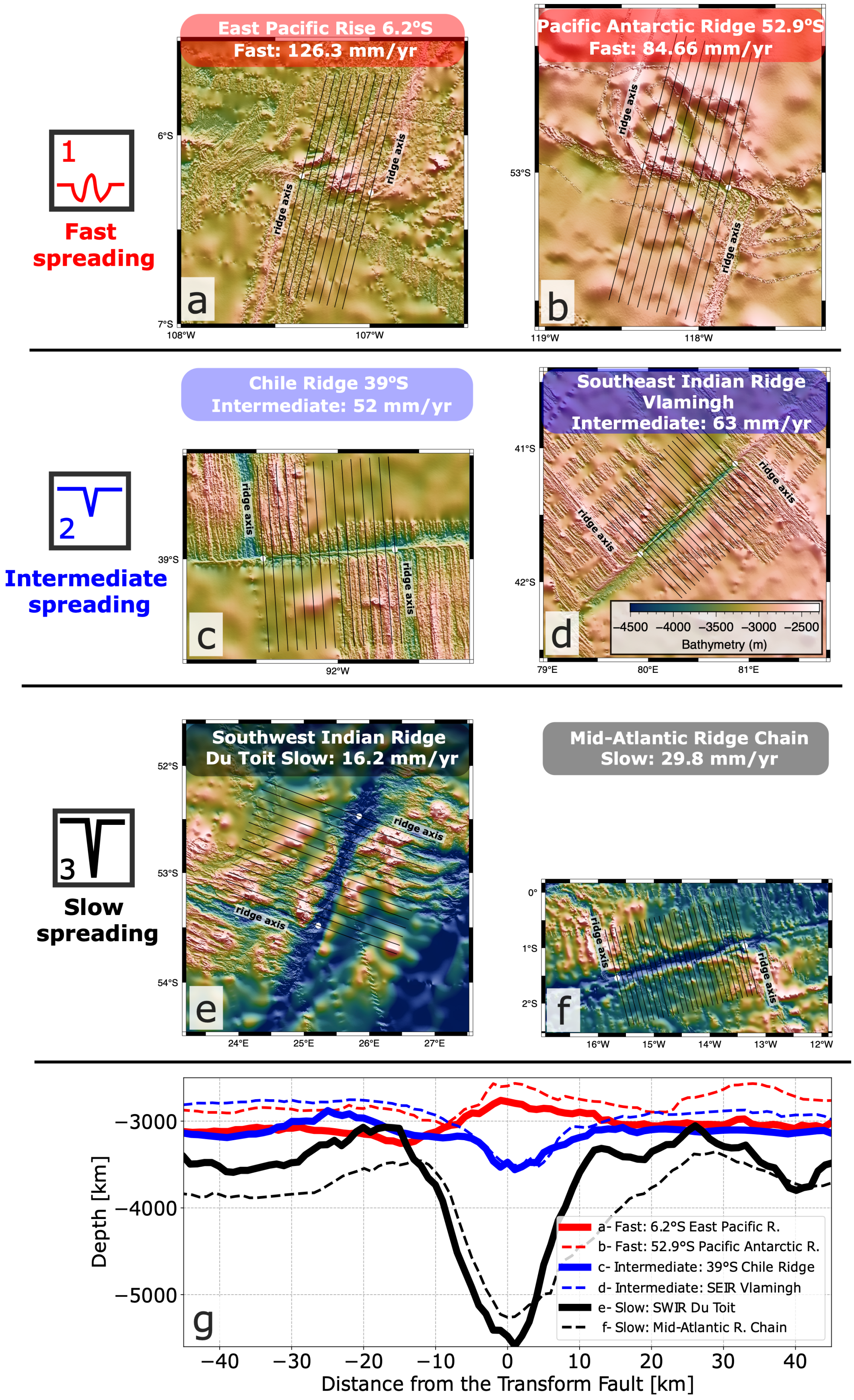

East Pacific Rise 6.2°S
Fast: 126.3 mm/yr
Pacific Antarctic Ridge 52.9°S
Fast: 84.66 mm/yr
1
Fast spreading
ridge axis
a
b
6°S
7°S
108°W
107°W
53°S
119°W
118°W
Chile Ridge 39°S
Intermediate: 52 mm/yr
Southeast Indian Ridge Vlamingh
Intermediate: 63 mm/yr
2
Intermediate spreading
c
d
39°S
92°W
41°S
42°S
79°E
80°E
81°E
−4500 −4000 −3500 −3000 −2500
Bathymetry (m)
Southwest Indian Ridge
Du Toit Slow: 16.2 mm/yr
Mid-Atlantic Ridge Chain
Slow: 29.8 mm/yr
3
Slow spreading
e
f
52°S
53°S
54°S
24°E
25°E
26°E
27°E
0°
1°S
2°S
16°W
15°W
14°W
13°W
12°W
g
Depth [km]
−3000
−4000
−5000
−40 −30 −20 −10 0 10 20 30 40
Distance from the Transform Fault [km]
a- Fast: 6.2°S East Pacific R.
b- Fast: 52.9°S Pacific Antarctic R.
c- Intermediate: 39°S Chile Ridge
d- Intermediate: SEIR Vlamingh
e- Slow: SWIR Du Toit
f- Slow: Mid-Atlantic R. Chain

Supplementary Fig. 1 | Additional examples for the characteristic bathymetry [1] and transform-perpendicular averaged bathymetric profiles for the 3 major modes of transform fault topographies. (**a, b**) Mode 1 ridge-like topographies at 6.2°S East Pacific Rise (EPR) transform fault with a full spreading rate of 126.3 mm/yr [2] and at 52.9°S Pacific Antarctic Ridge transform fault with a full spreading rate of 84.66 mm/yr [2]; (**c, d**) Mode 2 intermediate valleys at 39°S Chile Ridge with a full spreading rate of 52 mm/yr [3] and at 41.5°S Southeast Indian Ridge (SEIR) Vlamingh transform fault with a full spreading rate of 63 mm/yr [3]; and (**e, f**) Mode 3 deep valley at 53°S Southwest Indian Ridge (SWIR), Du Toit Transform Fault with a full spreading rate of 16.2 mm/yr [3] and at 1.2°S Mid-Atlantic Ridge (MAR), Chain Transform Fault with a full spreading rate of 29.8 mm/yr [3]. (**g**) Averaged across-transform topography for each mode shown in (a,b), (c,d) and (e,f).

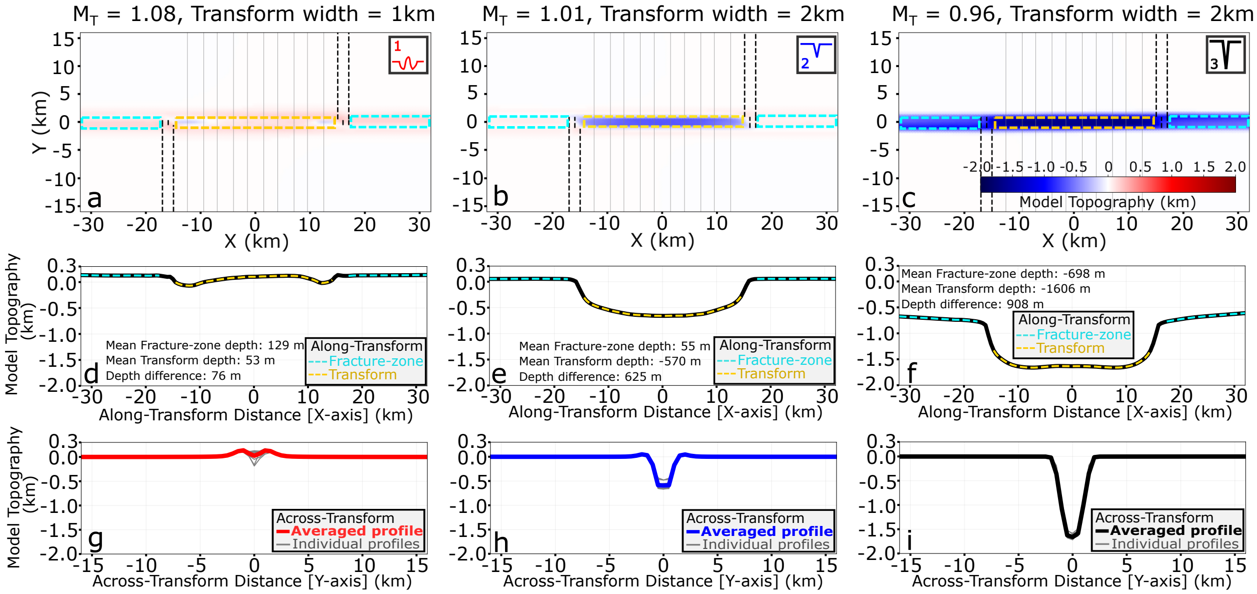

Supplementary Fig. 2 | **Averaged along- and across- transform model topographic profiles** for the three base model examples of Mode 1, 2, and 3 topographies shown in Fig. 3. (**a, b, c**) are map views of model topography for Modes 1, 2 and 3 respectively. (**d, e, f**) show the mean along- transform topographic profile for each case, with dashed cyan lines along the fracture-zones (highlighted with dashed cyan rectangles in a, b, c) and dashed yellow lines along the transforms (highlighted with dashed yellow rectangles in a, b, c). (**g, h, i**) show averaged across-transform topographic profiles from the 10 evenly spaced across-transform grey lines.

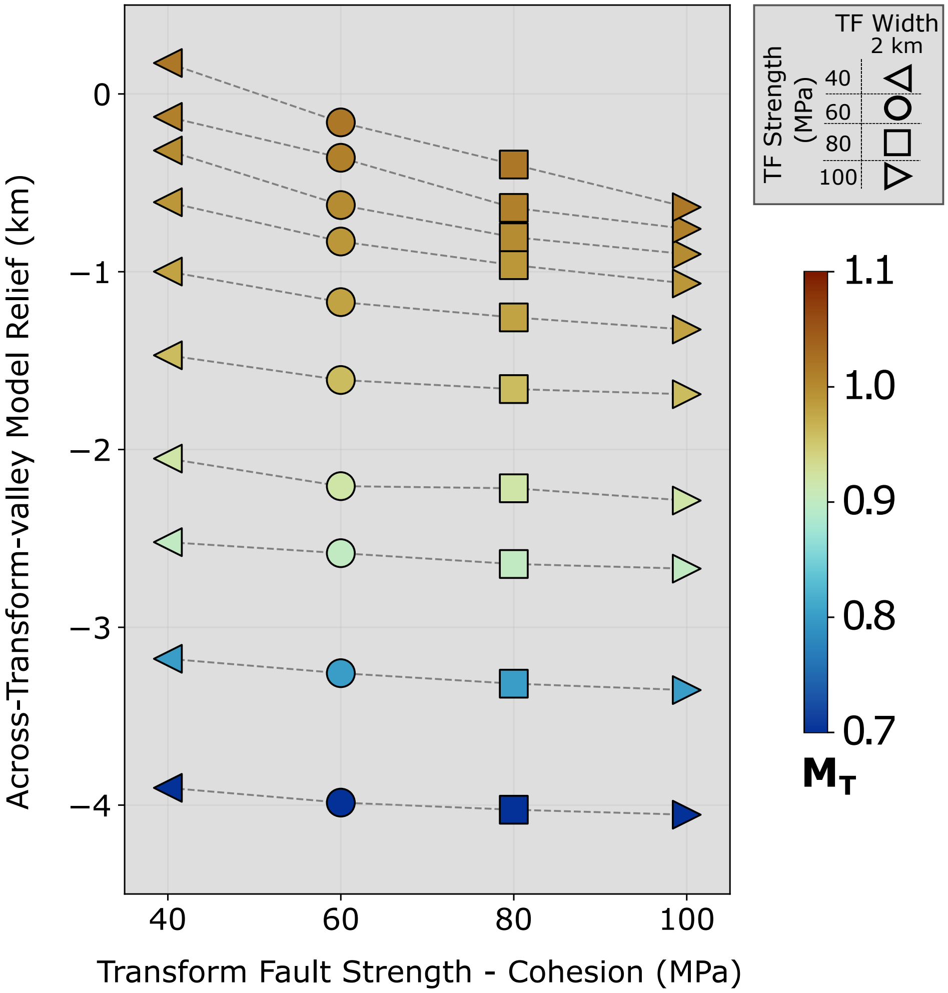

Supplementary Fig. 3 | Model across-transform valley relief as a function of M_T and transform fault (TF) strength varied the by different values of cohesion. Dashed lines illustrate trends of little or no dependence of transform valley depth on fault strength when far-field stretching dominates for the deep Mode 3 valleys (blue-green). By contrast, when there is little or no far-field tectonic extension ($M_T \approx 1$), shear-induced tension leads to low-relief valleys (Mode 2) (yellow-yellowish green), in which case transform valley depth increases with cohesion.

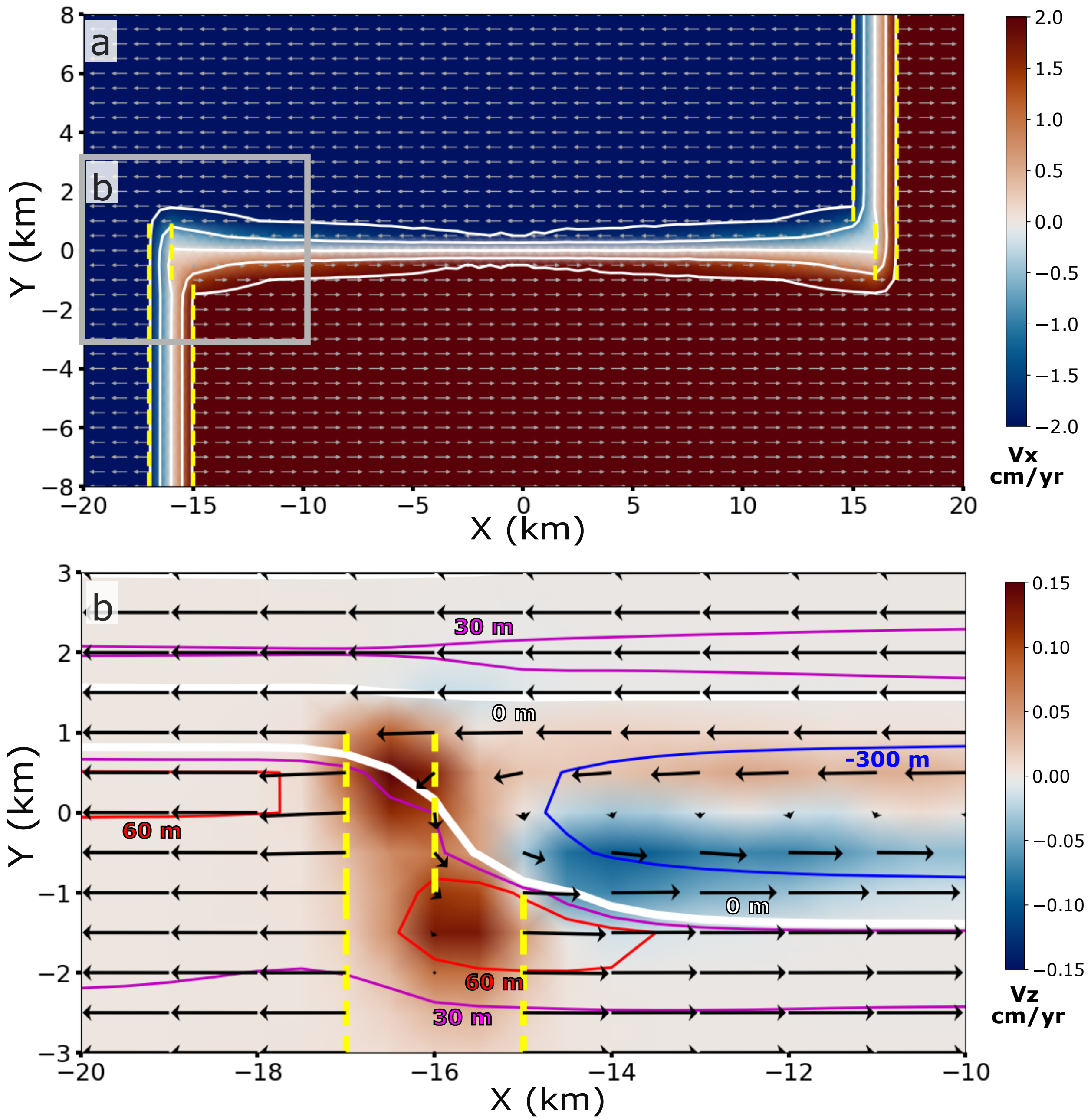

Supplementary Fig. 4 | Transform zone velocities near a dike. For Mode 2 example, (**a**) mapview velocity in X direction at 0.5 km depth. Yellow dashed lines mark the edges of the magma intrusion zones (dikes). White lines are velocity contours of Vx = +/-1.9, +/-1 and 0 cm/yr. Arrows are velocity vectors. The grey box indicates the extent of panel (**b**) which shows horizontal velocity vectors (length at X = -20 representing 2 cm/yr) overlay topographic contours of -300 m (blue lines), 0 m (white lines), 30 m (purple lines) and 60 m (red lines) and mapview of vertical velocity Vz.

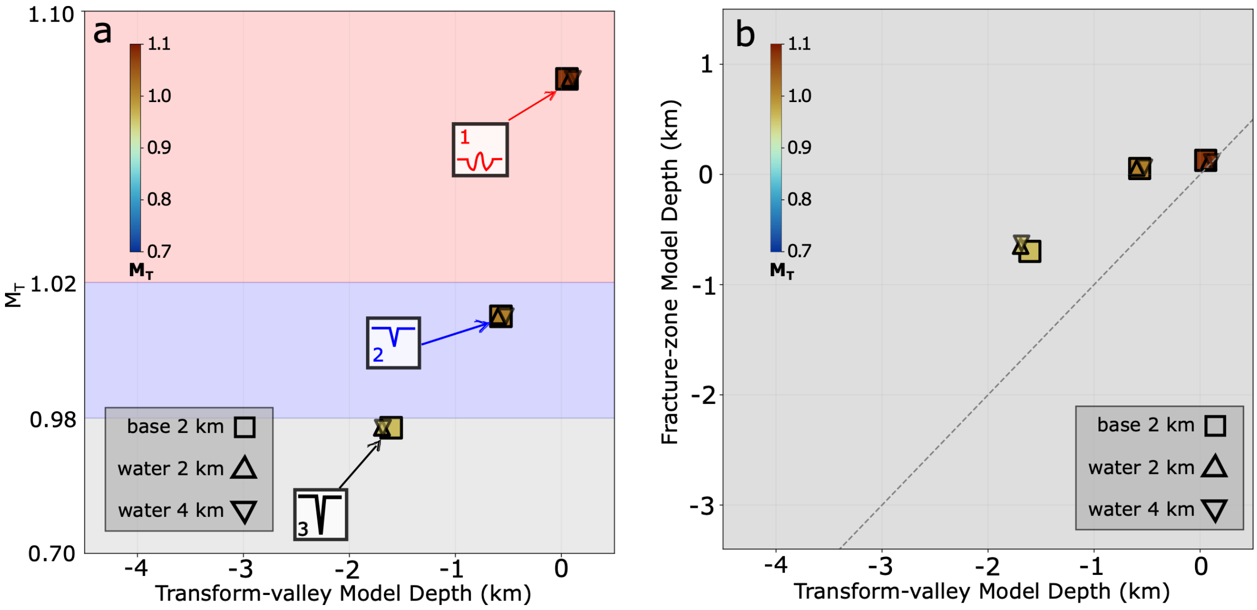

Supplementary Fig. 5 | Effect of seawater overburden pressure on transform valley and fracture zone depth. The 3 example base cases (rectangles) shown in Fig. 3, have negligible overburden pressure, but show very similar results to cases with 2 km (upward pointing triangles) or 4 km (downward pointing triangles) of ocean water (density of 1000 kg/m^3) overburden. Colors are for M_T. **a**) Transform valley model depth as a function of M_T as shown in Fig. 3d. **b**) Transform valley and fracture zone model depths as a function of M_T as shown in Fig. 4a (also see Supplementary Table 2 for details).

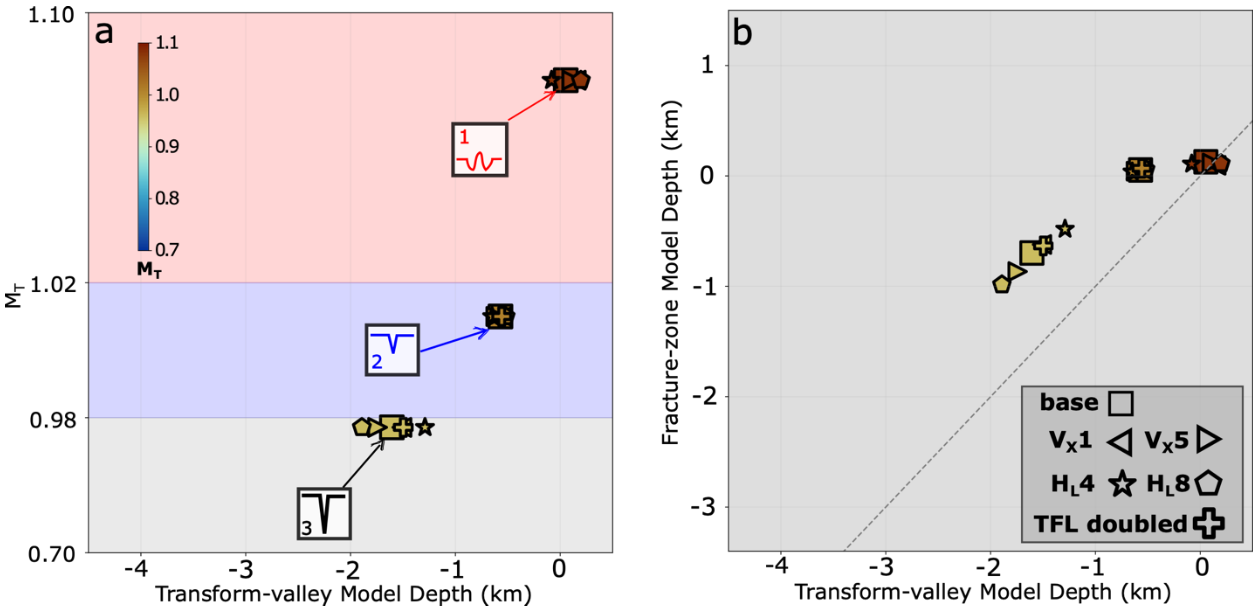

Supplementary Fig. 6 | Effect of spreading rate, lithospheric thickness and transform length on transform and fracture zone model depths. Symbol fill colors denote transform domain dike intrusion rate M_T. Rectangles are three base model examples shown in Fig. 3 that has half spreading rate of 2 cm/yr, lithospheric thickness of 6 km and transform fault length of 32 km. Triangles Vx1 (leftward pointing) and Vx5 (rightward pointing) indicate half spreading rates of 1 cm/yr and 5 cm/yr, respectively, keeping all other parameters the same as the base cases denoted with rectangles. H_L4 (stars) and H_L8 (pentagons) show results for lithospheric thickness of 4 km and 8 km, respectively. The crosses are for the 3 base cases, but with model domain in X and Y doubled and transform fault length (TFL) increases from 32 to 62 km. **a**) Transform valley model depth as a function of M_T as shown in Fig. 3d. **b**) Transform valley and fracture zone model depths as a function of M_T as shown in Fig. 4a (also see Supplementary Table 2 for details).

Supplementary Table 1. Model parameters and results shown in Fig. 3 & 4. Main controlling parameters are M_T and cohesion, and main model results are averaged fracture zone depth (“Avg FZ”) and averaged along- and across- transform fault depth (“Avg TF” and “Avg across-TF”) relative to the initial surface at 0 m, and depth difference between fracture zones and their adjacent transform (“FZ-TF”).

#	M_T	Cohesion [MPa]	Transform width [km]	Overburden density [kg/m3]	Vx [cm/yr]	H_Litho [km]	TF Length [km]	Avg FZ [m]	Avg TF [m]	FZ-TF [m]	Avg across-TF [m]
**1**	0.7	40	2	1	2	6	32	-2042	-3841	1799	-3903
2	0.8	40	2	1	2	6	32	-1543	-3119	1576	-3177
3	0.9	40	2	1	2	6	32	-1472	-2454	982	-2521
4	0.92	40	2	1	2	6	32	-1100	-2005	905	-2051
5	0.96	40	2	1	2	6	32	-622	-1413	791	-1470
6	0.98	40	2	1	2	6	32	-332	-965	633	-999
7	0.99	40	2	1	2	6	32	1	-585	586	-609
8	1	40	2	1	2	6	32	41	-280	321	-318
9	1.01	40	2	1	2	6	32	56	-17	73	-129
10	1.02	40	2	1	2	6	32	58	97	-39	174
11	1.02	40	1	1	2	6	32	72	-49	121	-103
12	1.04	40	1	1	2	6	32	95	65	30	125
13	1.06	40	1	1	2	6	32	109	141	-32	203
14	1.08	40	1	1	2	6	32	99	194	-95	260
15	1.1	40	1	1	2	6	32	131	226	-95	290
16	0.7	60	2	1	2	6	32	-2092	-3926	1834	-3986
17	0.8	60	2	1	2	6	32	-1545	-3188	1643	-3258
18	0.9	60	2	1	2	6	32	-1431	-2530	1099	-2583
19	0.92	60	2	1	2	6	32	-1115	-2155	1040	-2206
20	0.96	60	2	1	2	6	32	-670	-1558	888	-1609
21	0.98	60	2	1	2	6	32	-371	-1129	758	-1171
22	0.99	60	2	1	2	6	32	-58	-784	726	-830
23	1	60	2	1	2	6	32	38	-584	622	-625
24	1.01	60	2	1	2	6	32	61	-289	350	-359
25	1.02	60	2	1	2	6	32	69	-5	74	-160
26	1.02	60	1	1	2	6	32	66	-143	209	-205
27	1.04	60	1	1	2	6	32	95	17	78	-126
28	1.06	60	1	1	2	6	32	110	49	61	121
29	1.08	60	1	1	2	6	32	50	207	-157	280
30	1.1	60	1	1	2	6	32	147	192	-45	273
31	0.7	80	2	1	2	6	32	-2073	-3973	1900	-4026
32	0.8	80	2	1	2	6	32	-1606	-3233	1627	-3317
33	0.9	80	2	1	2	6	32	-1447	-2580	1133	-2644
34	0.92	80	2	1	2	6	32	-1050	-2165	1115	-2218
35	0.96	80	2	1	2	6	32	-698	-1606	908	-1660
36	0.98	80	2	1	2	6	32	-409	-1199	790	-1257
37	0.99	80	2	1	2	6	32	-194	-907	713	-963
38	1	80	2	1	2	6	32	7	-714	721	-806
39	1.01	80	2	1	2	6	32	54	-570	624	-641
40	1.02	80	2	1	2	6	32	73	-297	370	-397
41	1.02	80	1	1	2	6	32	62	-207	269	-322
42	1.04	80	1	1	2	6	32	94	-139	233	-236
43	1.06	80	1	1	2	6	32	139	-46	185	-182
44	1.08	80	1	1	2	6	32	128	53	75	135
45	1.1	80	1	1	2	6	32	59	231	-172	324
46	0.7	100	2	1	2	6	32	-2061	-3986	1924	-4054
47	0.8	100	2	1	2	6	32	-1631	-3260	1629	-3353
48	0.9	100	2	1	2	6	32	-1448	-2599	1151	-2670
49	0.92	100	2	1	2	6	32	-1112	-2221	1109	-2287
50	0.96	100	2	1	2	6	32	-755	-1629	874	-1689
51	0.98	100	2	1	2	6	32	-475	-1248	773	-1325
52	0.99	100	2	1	2	6	32	-247	-986	739	-1066
53	1	100	2	1	2	6	32	-80	-788	708	-901
54	1.01	100	2	1	2	6	32	29	-659	688	-759
55	1.02	100	2	1	2	6	32	63	-535	598	-637
56	1.02	100	1	1	2	6	32	49	-244	293	-396
57	1.04	100	1	1	2	6	32	84	-199	283	-346
58	1.06	100	1	1	2	6	32	108	-128	236	-251
59	1.08	100	1	1	2	6	32	128	-31	159	-153
60	1.1	100	1	1	2	6	32	137	66	71	155

Supplementary Table 2.

Model parameters and results for sensitivity tests. Tested parameters are highlighted with bold fonts and are underlined. For models with transform length of 62 km, model domain size in X and Y axes are doubled to 128 and 64 km.

#	M_T	Cohesion [MPa]	Transform width [km]	Overburden density [kg/m³]	Vx [cm/yr]	H_Litho [km]	Transform Length [km]	Avg FZ [m]	Avg TF [m]	FZ-TF [m]
BASE MODE1	**1.08**	80	1	1	2	6	32	128	53	75
	1.08	80	1	**1000 (2km)**	2	6	32	111	79	32
	1.08	80	1	**1000 (4km)**	2	6	32	114	109	5
	1.08	80	1	1	**1**	6	32	96	153	-57
	1.08	80	1	1	**5**	6	32	117	111	5
	1.08	80	1	1	2	**4**	32	109	-82	191
	1.08	80	1	1	2	**8**	32	112	197	-85
	1.08	80	1	1	2	6	**62**	-162	637	-799
BASE MODE2	**1.01**	80	2	1	2	6	32	54	-570	624
	1.01	80	2	**1000 (2km)**	2	6	32	59	-598	657
	1.01	80	2	**1000 (4km)**	2	6	32	65	-525	590
	1.01	80	2	1	**1**	6	32	57	-550	607
	1.01	80	2	1	**5**	6	32	43	-614	657
	1.01	80	2	1	2	**4**	32	37	-649	686
	1.01	80	2	1	2	**8**	32	60	-521	581
	1.01	80	2	1	2	6	**62**	69	-558	627
BASE MODE3	**0.96**	80	2	1	2	6	32	-698	-1606	908
	0.96	80	2	**1000 (2km)**	2	6	32	-650	-1693	1043
	0.96	80	2	**1000 (4km)**	2	6	32	-625	-1684	1059
	0.96	80	2	1	**1**	6	32	-621	-1510	889
	0.96	80	2	1	**5**	6	32	-866	-1742	875
	0.96	80	2	1	2	**4**	32	-481	-1290	809
	0.96	80	2	1	2	**8**	32	-985	-1892	907
	0.96	80	2	1	2	6	**62**	-634	-1497	863

Supplementary Discussion

1. Sensitivity tests

Globally, oceanic transform faults are associated with different spreading rates, fault lengths, seafloor depths, and lithospheric thicknesses. To test the robustness of our main conclusion that transform domain magmatism plays a first-order role in controlling transform topography, and to gauge the parameter space over which our results are applicable, we investigated the effects of different ocean water depths, half spreading rates, lithospheric thicknesses, and transform lengths on transform and fracture zone morphologies (Supplementary Fig. 5 & 6, Supplementary Table 2).

1.1 Sensitivity to ocean water overburden

The base models presented in Fig. 3 & 4 assumed a 2-km "sticky-air" layer with a density of 1 kg/m^3, which results in negligible overburden pressure (< 0.1% as compared to when using 3 km of seawater) onto the modeled internal free-surface. In reality, the ocean water pressure on the seafloor increases with deeper seafloor at slower spreading rate mid-ocean ridges (Fig. 3e). Hence, starting with the models shown in Fig 3, we vary the sea water overburden and quantify its effects on transform valley and fracture zone depths (Supplementary Fig. 5). With 2 km of water (with an assumed density of 1000 kg/m^3) overlying the model seafloor, the Mode 1 model (Fig. 3a) shows little change. The Mode 2 case (Fig. 3b) shows a 28 m deeper transform valley that changes from -570 m to -598 m relative to the initial surface at 0 m, and 4 m shallower fracture zones that changes from 54 m to 59 m relative to the initial surface at 0 m. The Mode 3 model (Fig. 3c) has 48 m of shallower fracture zones that changes from -698 m to -650 m but an 87 m deeper transform valley that changes from -1606 m to -1693 m. With 4 km of overlying water, the results are very similar to that of the 2 km cases (Supplementary Fig. 5).

Overall, these differences are significantly smaller than the uncertainties in the observed data (error bars in Fig. 3e and Fig. 4b), which have averaged root mean square deviations of 614 m and 450 m for the measurements of the transform valley and fracture zone depths, respectively [3]. The limited influence of variable ocean water overburden on transform and fracture zone topography allows us to assume a mean seafloor depth of 3 km [4–6] when comparing the model transform and fracture zone depths with the observations (Fig. 3 & 4).

1.2 Sensitivity to variable half spreading rates

The models shown in Figs. 3 & 4 assume a half spreading rate *Vx* of 2 cm/yr; however, natural mid-ocean ridge systems have spreading rate that range from less than 1 cm/yr up to ~7 cm/yr (Fig. 3e). Hence, starting with the models shown in Fig. 3 a, b and c, we change the half spreading rate to 1 cm/yr and 5 cm/yr and compare predicted topography (Supplementary Fig. 6). Changing spreading rate alone does not alter the morphological mode of a model with a given value of M_T. For the Mode 1 and Mode 2 cases, when half spreading rate is either decreased to 1 cm/yr or increased to 5 cm/yr, negligible changes can be identified in transform valley and fracture zone model depths (Supplementary Fig. 6 and Table 2). For the Mode 3 case, increasing half spreading rate from 2 cm/yr to 5 cm/yr causes the average fracture zone depth to deepen by 168 m from -698 m to -866 and the average transform-valley depth to deepen by 136 m from -1606 m to -1742 m. Decreasing half spreading rate from 2 cm/yr to 1 cm/yr causes the average fracture zone model depth to become shallower by 77 m from -698 m to -621 m and the average transform-valley model depth to become shallower by 96 m from -1606 m to -1510 m. Even though faster spreading rates lead to deeper fracture zones and transform valleys for the Mode 3 cases, depth differences between transform and fracture zones remain similar and data clusters

around the same trend (Supplementary Fig. 6b). Also, these variations are less than one-third of the aforementioned uncertainties in the data.

1.3 Sensitivity to variable lithospheric thickness

The base models shown in Figs. 3 & 4 assumed a lithospheric thickness of 6 km, which is subject to change as a function of seafloor spreading rate. Hence, starting with the 3 cases in Fig. 3 a, b and c, we alter lithospheric thickness and quantify the effects on transform and fracture zone model topography (Supplementary Fig. 6). Again, Mode 1 and Mode 2 cases show negligible changes, but for the Mode 3 case, thicker lithosphere leads to deeper fracture zones and transform-valleys. When lithospheric thickness is increased from 6 km to 8 km, average fracture zone model depth becomes deeper by 287 m (from -698 m to -985) and the average transform-valley model depth becomes deeper by 286 m (from -1606 m to -1892 m). When lithospheric thickness decreases from 6 km to 4 km, average fracture zone model depth becomes shallower by 217 m (from -698 m to -481 m) and the average transform-valley model depth becomes shallower by 316 m (from -1606 m to -1290 m). The depth differences between the transform valley and fracture zone are almost invariant (Supplementary Fig. 6b) and the data clusters around the same trend as the main models (Fig. 4a). The changes are also less than the measured uncertainties in the data.

1.4 Sensitivity to transform fault length

Finally, to test the sensitivity to transform fault length, we increase the transform length from the value of 32 km used in the models shown in Fig. 3 a, b and c to 62 km (crosses in Supplementary Fig. 6). To keep the aspect ratio between the transform fault and ridge segment the same, we doubled the model domain in the X and Y directions. The Mode 1 case is the most time-dependent, with the longer transform leading to higher transform topography by 584 m

from 53 to 637 m and deeper fracture zones by 290 m from 128 m to -162 m. For the Mode 2 case, the longer transform results in negligible changes. For the Mode 3 case, the longer transform result in a shallower fracture zone and transform by 64 m (from -698 m to -634 m) and by 109 m (from -1606 m to -1497 m), respectively. The depth difference between the transform valley and fracture zone remains almost unchanged.

Note that the intermediate spreading rate data point (with a fracture zone depth of 1.96 km and transform depth of 2.98 km) that deviates from the model results in Fig. 4b is from the South East Indian Ridge at 78.4 °E, 38.55 °S. In this location average seafloor has been elevated by ~ 1 km as compared to the Vlamingh transform fault just southeastern (80.36 °E, 41.47 °S) to it due to the Amsterdam-St. Paul hotspot anomaly (e.g. ref [7]).

In summary, these sensitivity tests show that ocean water depth, half spreading rate, lithospheric thickness, and transform fault length play second-order roles in controlling the transform and fracture zone topography and in all cases their effects are smaller than the uncertainties in data measurements. These results support the first-order control of transform magmatism on modes of oceanic transform and fracture zone topography.

Supplementary References:

1. Ryan, W. B. *et al*. Global multi-resolution topography synthesis. *Geochemistry, Geophysics, Geosystems* **10**, (2009).
2. Kreemer, C., Blewitt, G. & Klein, E. C. A geodetic plate motion and Global Strain Rate Model. *Geochemistry, Geophysics, Geosystems* **15**, 3849–3889 (2014).
3. Grevemeyer, I., Rüpke, L. H., Morgan, J. P., Iyer, K. & Devey, C. W. Extensional tectonics and two-stage crustal accretion at oceanic transform faults. *Nature* **591**, 402–407 (2021).

4. Dalton, C. A., Langmuir, C. H. & Gale, A. Geophysical and geochemical evidence for deep temperature variations beneath mid-ocean ridges. *Science* **344**, 80–83 (2014).
5. Richards, F. D., Hoggard, M. J., Cowton, L. R. & White, N. J. Reassessing the thermal structure of oceanic lithosphere with revised global inventories of basement depths and heat flow measurements. *Journal of Geophysical Research: Solid Earth* **123**, 9136–9161 (2018).
6. Stein, C. A. & Stein, S. A model for the global variation in oceanic depth and heat flow with lithospheric age. *Nature* **359**, 123–129 (1992).
7. Ballmer, M. D., van Keken, P. E. & Ito, G. Hotspots, large igneous provinces, and melting anomalies. in (Elsevier, 2015).
